# Comprehensive strategies to defeat *Candida auris –* challenge and insight: A review

**DOI:** 10.1097/MD.0000000000046610

**Published:** 2025-12-19

**Authors:** Jieyu Zhang, Lichao Zhong, Chunxiang Chen, Licong Ye, Fei Cao, Hua Xia, Yi Hong

**Affiliations:** a Fuzhou Medical College of Nanchang University, Fuzhou, Jiangxi, China.

**Keywords:** antifungal, *Candida auris*, combination, novel strategies, prevention

## Abstract

*Candida auris*, an emerging fungus that exhibits a unique transmission ability, causes large healthcare-associated outbreaks on 6 continents since first reported in 2009. Most isolates have acquired fluconazole resistance, and resistance to other agents/classes emerges during drug exposure. The emergence of *C auris* infections raises serious concerns for public health. Systematic elaboration on clinical treatment and prevention of *C auris* infections is rare. We aim to summarize the research progress on the risk factors, clinical signs, drug treatment and prevention of *C auris* infections.

## 1. Introduction

In the last decades, fungal infections have been growing faster, presenting a serious threat to the global population, due to the fact that there are much fewer drug classes available than bacterial diseases.^[1,2]^ Since its first identification in Japan about 14 years ago (in 2009), *Candida auris* has broken out and spread in many regions around the world, such as Brazil, America, India, Mexico, and Europe,^[3–7]^ named after their original regions of discovery: clade I (South Asia), clade II (East Asia), clade III (South Africa), clade IV (South America), and clade V (Iran).^[8–12]^ A systematic review (2020) of nearly 5000 cases of *C auris* from 33 countries reported the overall crude mortality rate of *C auris* infection as high as 78%.^[13]^ Similar findings have been reported in the United States. The US hospital database from 2017 to 2022 revealed that the crude mortality rate was 47% for *C auris* bloodstream infection.^[14]^ Another frightening news from Mexico showed that mortality was extremely high (>83%) for patients with COVID-19-associated *C auris* bloodstream infection, even with antifungal therapy.^[15]^

The most worrisome aspect associated with *C auris* infection can be summarized as a high rate of antifungal drug resistance. Although, the commonly used antifungals for *C auris* include azoles, amphotericin B and echinocandin, of which echinocandin is the first-line treatment for invasive *Candida* infections, an antifungal susceptibility testing of 296 *C auris* isolates using population genomic analyses showed that 80% were fluconazole-resistant, 23% to were amphotericin B resistant, and 7% isolates were micafungin resistant.^[16]^

**Figure 1.**
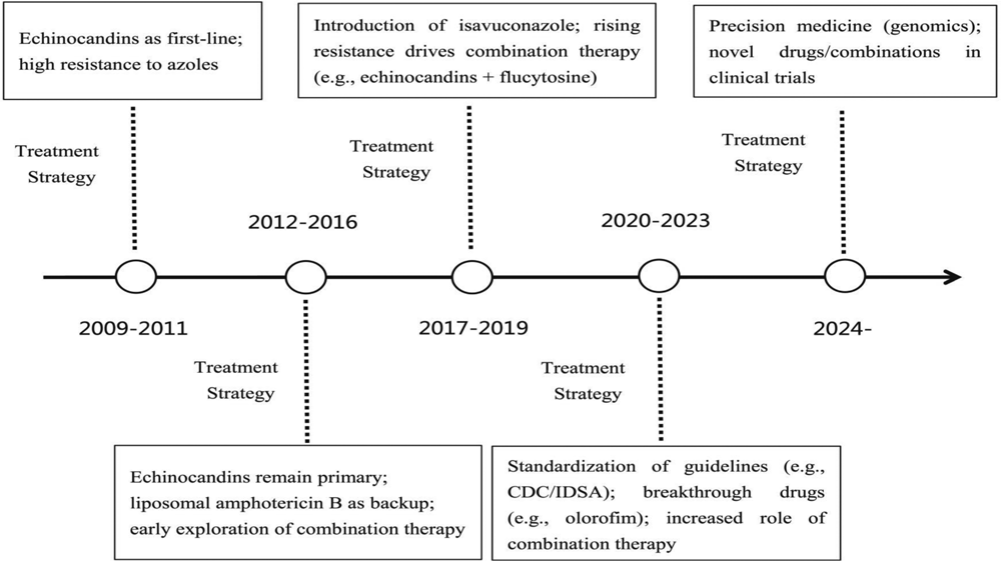
Timeline of treatment strategies for *Candida auris* infections. CDC = Centers for Disease Control and Prevention, IDSA = Infectious Diseases Society of America.

Most notably, the resistance of *C auris* to these drugs has increased in recent years. The US surveillance of *C auris* resistance identified an approximately 3-fold increase in the number of echinocandin-resistant *C auris* cases in 2021 compared to 2019, and an approximately 7% increase in resistance to azoles in 2019 to 2020.^[17]^ The treatment strategies for *C auris* infections have undergone continuous refinement in response to evolving antifungal resistance patterns and advancing clinical understanding (Fig. 1).

To address the challenges posed by *C auris* infections to global public health, this review aimed to summary the risk factors and new therapeutic strategies to fight *C auris*.

## 2. Materials and methods

### 2.1. Ethics

Ethics application was not required as all research data in this study comes from publicly available professional literature.

### 2.2. Data sources

We searched the Cochrane Library, PubMed, Medline, Embase, and China National Knowledge Infrastructure to identify relevant articles on *C auris* published before June 2024 without restriction of language. The following keywords were used: *C auris*. Two researchers reviewed and screened the titles, abstracts and the full text. Articles were deduplicated and excluded if there was no, or passing, reference to *C auris.* Data were extracted from eligible studies including name of the first author, publication year, study design, the risk factors, clinical signs, drug treatment and prevention of *C auris* infections.

## 3. Results

A total of 114 results were available for inclusion in the review. The findings were thematically grouped and were presented below.

### 3.1. Risk factors and clinical signs

*C auris* is an emerging fungal pathogen notorious for its persistent transmission in healthcare settings, particularly among high-risk patient populations.^[18]^ The risk factors for *C auris* infection are similar to other *Candida* species.^[19]^ Demographic analyses indicate increased susceptibility among male patients and individuals aged ≥58 years.^[20]^ A retrospective analysis of 912 patients with *C auris* infection between 2009 and 2020 revealed the 5 most prevalent risk factors, namely recent broad-spectrum antibiotic exposure, utilization of central venous catheters, intensive care units, indwelling urinary catheter use, and surgery.^[20]^ Multisite colonization, mechanical ventilation,^[21]^ parenteral nutrition,^[21]^ immunocompromising diseases (diabetes mellitus, malignancy, chronic kidney disease, neutropenia)^[21,22]^, and other *Candida* infections^[22,23]^ are also related risk factors for *C auris* infection. The dissemination of this resistant fungal pathogen is complex and multifactorial but climatic changes, delay to report *C auris*, lapses in hand hygiene for health care workers, deficiencies in implementation of infection prevention and control (IPC) measures, and inadequate disinfection of the patient equipment such as ventilators and environment might also have a role^[24–26]^ (Table 1).

**Table 1:** The risk factors for *C auris* infection.

Author	Risk factor	OR (95% CI)	References
Briano	Multisite colonization	9.45 (1.28–70.00)	^[3]^
Hu	Broad-spectrum antibiotic therap	–	^[20]^
	Utilization of central venous catheters	–	
	Employment of urinary catheters	–	
	Surgery	–	
	Male	–	
	Over 58 yr old	–	
Tian	Diarrhea	12.25 (2.11–70.99)	^[21]^
	Gastrointestinal decompression	19.33 (2.05–182.55)	
	Infection, or colonization with other Candida isolates	7.00 (1.17–42.00)	
	Tetracycline antibiotics	25.38 (2.71–237.6)	
Garcia-Bustos	Total parenteral nutrition	3.73	^[22]^
	Rectal isolate	1.81	
	Sepsis	1.75	
	Arterial catheter	1.46	
	Parenteral nutrition	1.32	
Barantsevich	Other Candida infections	–	^[23]^
	Broad-spectrum antibiotic therap	–	
Hofer	Climatic changes	–	^[24]^
Allaw/prestel	Deficiencies in implementation of infection prevention and control (IPC) measures	–	^[25,26]^
Proctor	Multisite colonization	–	^[27]^

CI = confidence interval, IPC = infection prevention and control, OR = odds ratios.

*C auris* has a particular predilection for the skin, particularly the axilla and groin.^[27]^ Urinary tract colonization is relatively uncommon but can occur in the presence of specific risk factors, such as an indwelling urinary catheter. *C auris* infection most commonly presents with candidemia, but also present with symptoms in wounds, central nervous system, respiratory tract, abdomen, bone, soft tissues and other parts of the body.^[28–31]^ In rare cases, *C auris* infection can also induce symptoms such as endophthalmitis, liver and spleen abscess, intestinal perforation.^[32]^

Overall, the increasing resistance of *C auris* to antifungal agents and the growing number of people with underlying diseases, as well as clinical manifestations ranging from candidemia to an array of highly invasive and life-threatening clinical syndromes are likely to elevate morbidity and mortality in the population, increase the difficulty of curing *C auris* infections, and pose a serious risk to clinical management. The current epidemiological landscape underscores an urgent need for novel therapeutic strategies to combat the rapid emergence of drug-resistant *C auris* strains and mitigate this growing public health threat.

### 3.2. Antifungal agents in clinical development against C auris

Currently, several new antifungal agents have been entered into clinical studies, including opelconazole, VT-1598, rezafungin, ibrexafungerp, fosmanogepix, manogepix and T-2307, and their characteristics are summarized in Table 2.

**Table 2:**
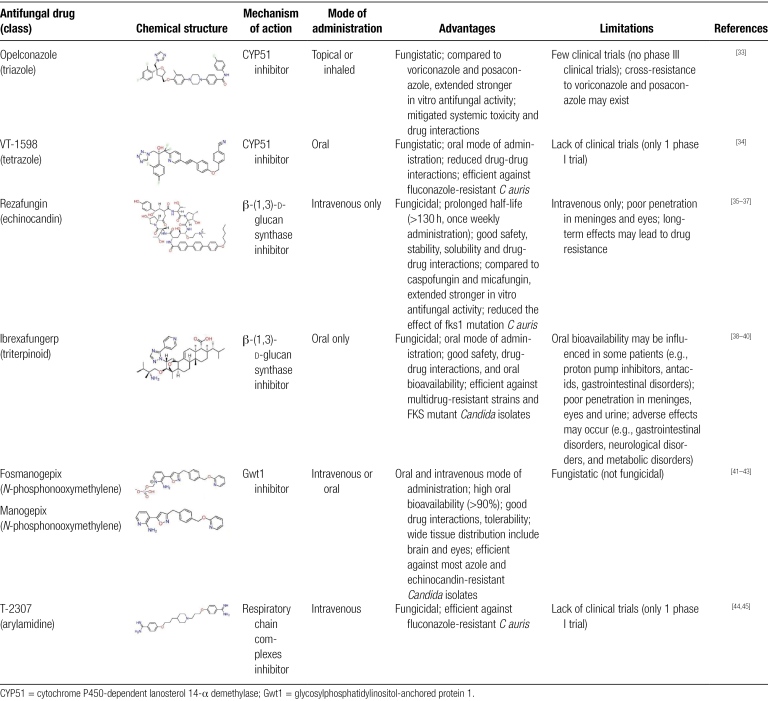
Mechanism of action, advantages and limitations of antifungal drugs in clinical development against *C auris*.

#### 3.2.1. Opelconazole (formerly PC945) and VT-1598

Azoles interfere with ergosterol synthesis and disrupt fungal cell membranes by inhibiting fungal 14-α-demethylase (CYP51).^[46]^ Opelconazole, a novel triazole optimized for inhalation or topical treatment, showed stronger antifungal activity against *C auris* from clades I to IV than voriconazole and posaconazole in vitro, with the geometric mean minimum inhibitory concentration (MIC), MIC_50_ and MIC_90_ values of 0.058, 0.063, and 0.25 µg/mL. In addition, it may mitigate systemic toxicity and drug interactions.^[33]^ Another tetrazole VT-1598, also exhibited antifungal activity in vitro against 100 clinical strains and resistant strains of *C auris* from clades I to IV, with MIC values of 0.03 to 8 µg/mL. Preclinical validation in a neutropenic murine model confirmed VT-1598’s therapeutic potential, showing significant reductions in fungal burden in both kidney and brain tissues, accompanied by improved survival outcomes compared to control groups.^[34]^

#### 3.2.2. Rezafungin

Echinocandins disrupt the cell wall of the fungus by inhibiting the synthesis of β-(1,3)-d-glucan.^[35]^ However, mutations in the fks gene can confer reduced echinocandin susceptibility in *C auris*.^[47]^ Rezafungin, a novel echinocandin, showed potent antifungal activity against 122 isolates of *C auris* from India, with the MIC values ranging from 0.016 to 16 µg/mL, MIC_50_ of 0.25 µg/mL, and MIC_90_ of 1 µg/mL, and its antifungal activity was better than caspofungin and micafungin. In addition, fks1 mutant strains of *C auris* exhibit significantly smaller MIC elevations with rezafungin compared to anidulafungin and micafungin.^[36]^ In a mouse model infected with *C auris*, 20 mg/kg rezafungin was effective in reducing fungal burden.^[36]^ Clinical evaluation through the phase II trial (NCT02734862) has confirmed both the safety and therapeutic potential of intravenous rezafungin.^[37]^ Moreover, rezafungin offers distinct pharmacokinetic advantages, including an extended half-life (>130 hours) enabling once weekly intravenous administration, along with enhanced stability and solubility that simplify treatment regimens.

#### 3.2.3. Ibrexafungerp (IBX, formerly SCY-078)

Ibrexafungerp (IBX), a novel triterpenoid antifungal agent, is an orally active inhibitor of glucan synthase with good safety, oral availability and excellent tissue distribution. IBX demonstrates low MIC values against *C auris* isolates from all geographical clades (I–IV) in vitro. In vitro, IBX showed potent antifungal activity against both multidrug-resistant strains and fks mutant strains, with the MIC values ranging from 0.06 to 2 µg/mL, MIC_50_ of 0.5 µg/mL and MIC_90_ of 1 µg/mL.^[38,39]^ In vivo, oral IBX administration significantly reduced fungal burden in the kidneys of *C auris*-infected mice.^[39]^ Clinical data from phase II and III trials further support its efficacy in treating invasive candidiasis, highlighting its potential as a broad-spectrum oral antifungal option.^[38–40]^

#### 3.2.4. Manogepix (APX001A, E1210) and fosmanogepix (APX001, E1211)

As a highly promising, next-generation antifungal agent effective against all clades of *C auris*, manogepix (MGX), the active metabolite of fosmanogepix (FMGX), exhibits a unique antifungal mechanism that targeted glycosylphosphatidylinositol (GPI)-anchored wall transfer protein 1 (Gwt1), impairs the maturation and localization of mannoproteins, affects GPI synthesis and ultimately disrupts the *C auris* cell wall and cell membrane.^[41]^ In vitro, the MIC values of fosmanogepix against 394 *C auris* isolates ranged from 0.002 to 0.063 µg/mL, MIC_50_ was 0.008 µg/mL and MIC_90_ values was 0.016 µg/mL, which were measured by the broth microdilution (BMD) method.^[41]^ In vivo, fosmanogepix significantly reduced fungal burden in the kidneys and brains of infected mice and improved survival rates in systemic infection models.^[42,43]^ In addition, fosmanogepix can be switched between intravenous and oral formulations without affecting blood concentrations due to its high oral bioavailability, good tolerability, and favorable tissue penetration.^[42,43]^ Clinical data from a phase II trial further support its therapeutic potential, demonstrating an 80% treatment success rate at the end of therapy.^[43]^

#### 3.2.5. T-2307

Similar to ibrexafungerp and fosmanogepix, T-2307 demonstrates potent in vitro activity against all known clades (I–IV) of *C auris*, including multidrug-resistant strains. The arylamines T-2307 selectively inhibit respiratory chain complexes, disrupt mitochondrial cell membrane potential, and impair mitochondrial function.^[44]^ In vitro, T-2307 showed MICs ≤ 0.008 to 0.015 µg/mL against 23 clinical isolates of *C auris*, including fluconazole-resistant strains.^[44]^ In murine models of *C auris* infection, a dose of 3 mg/kg/d significantly improved survival rates.^[44]^ Notably, T-2307 has successfully completed phase I clinical trials with a favorable safety profile and no reported adverse effects, supporting its potential for further development.^[45]^

### 3.3. Novel drugs in preclinical development against C auris

Current strategies for antifungal drug development focus on multiple approaches, including: targeted inhibition of fungal-specific pathways, synthesizing new compounds, screening existing compound libraries, and immunomodulation to enhance host defense mechanisms. As demonstrated in this review, most investigational antifungals exhibit potent in vitro and in vivo activity against *C auris*, with their spectrum of activity and potency profiles comprehensively summarized in Table 3.

**Table 3:** Mechanism of action and in vitro antifungal activity (MIC) of novel drugs in preclinical development against *C auris*.

Class	Drug	*C auris* origin/clades (samples)	Mechanism of action	MIC (µg/mL)	References
Antibodies	*C.aur*-lgG	*C auris* CDC0389 (n = 1)	Boosted immunity	–	^[48]^
	MAbs C3.1	*C auris* CAU-06 and CAU-09 (n = 2)	β-Man3 inhibitor	–	^[49]^
	MAbs 6H1		Hwp1 inhibitor	–	
	MAbs 9F2		Pgk1 inhibitor	–	
	Anti-HILp MAb	Clades I–IV (n = 5)	*Cau*-HILp epitope inhibitor	–	^[50]^
Sec14p inhibitor	Turbinmicin	*C auris* B11220 (n = 3)	Targeted the Sec14p protein	0.125	^[51,52]^
Benzoanilide antifungal drugs	A1	*C auris* 0029, 0030, and 15,448 (n = 3)	GPI-anchored proteins inhibitor	0.06–2.0	^[53]^
Antifungal peptide	Dimer 1	*C auris* DSMZ-No. 21092 (n = 3)	Formed pores or related activities, disrupted the functional integrity of the cell wall	–	^[54,55]^
	Dimer 2			–	
	LL-37	Clade III (n = 10)	Arrested the cell cycle in S phase	25–100	^[56]^
Nanoparticles	AgNPs	Clades I–IV (n = 10)	Affected molecular targets, disrupted cellular biofilms and ultrastructure, and damaged the fungal structure and metabolism	<0.5	^[57,58]^
	BiNPs	Clades I–IV (n = 10)	Disrupted the structure of fungal biofilms and cellular morphology	1–4	^[59]^
	Ag–Cu–Co NPs	Clade III (n = 25)	Arrested the cell cycle in G2/M phase	0.19–0.39	^[60]^
	Ag–Fe NPs	Clade III (n = 25)	Arrested the cell cycle in G2/M phase	0.39–0.78	^[61]^
Antidepressant agent (repositioning drugs)	Sertraline	*C auris* 70, 33 and IL (n = 3)	CYP51 inhibitor	20–40	^[62]^
Derivatives of mefloquine (repositioning drugs)	2450	*C auris* 0383, 0385, 0387–0389 (n = 5)	Interfered with mitochondrial and vacuolar function	2–8	^[63]^
	4377				
	13,480				
	3,05,758				
Aldehyde dehydrogenase enzyme inhibitor (repositioning drugs)	Disulfiram	*C auris* CBS10913 and *C auris* CBS12373 (n = 2)	Increased cell aggregation and decreased biofilm formation	1–8	^[64]^
Piperidine based 1,2,3-triazolylacetamide derivatives	Pta1-Pta3	Clade III (n = 25)	Arrested the cell cycle in S phase	0.24–0.97	^[65]^
Cinnamaldehyde-based azole derivatives	6f	Clade III	Arrested the cell cycle in S phase and G2/M phase	0.98	^[66]^
Eugenol tosylate congeners	C5	Clade III (n = 5)	Arrested cell cycle in G0/G1 phase	0.98	^[67]^

n: the number of *Candida auris* samples used in the experiment.

β-(Man)3 = β-1,2-mannotriose, Ag–Cu–Co NPs = Ag–Cu–Co trimetallic nanoparticles, Ag–Fe NPs = Ag–Fe bimetallic nanoparticles, *C.aur*-lgG = antibodies (lgG) against *Candida auris*, Cau-HILp = *Candida auris* Hyr1p/Iff-like protein, CYP51 = cytochrome P450-dependent lanosterol 14-α demethylase, GPI = glycosylphosphatidylinositol, Gwt1 = glycosylphosphatidylinositol-anchored protein 1, Hwp1 = hyphal wall protein 1, MIC = minimum inhibitory concentration, Pgk1 = phosphoglycerate kinase 1.

#### 3.3.1. Antibodies

Doron I et al^[48]^ found that intestinal fungal colonization induces germinal center-dependent B cell expansion and systemic antibody (*C.aur*-lgG) production in extraintestinal lymphoid tissues. When *C auris* was monocolonized in the intestine of germ-free mice, the mice produced the IgG antibodies (*C.aur*-lgG). The *C.aur*-IgG antibodies demonstrated significant protective efficacy, conferring immunity against systemic *C auris* infection and reducing mortality rates in challenged mice. In addition, *C.alb*-IgG antibody against *C albicans* also protected mice infected with *C auris*, suggesting that these antibodies have a certain degree of cross-protection between the same genera of fungi.

In A/J mouse model, monoclonal antibodies (mAbs) C3.1, 6H1 and 9F2 targeted cell surface antigens β-1,2-mannotriose (β-Man3) and hyphal wall protein 1 (Hwp1) and phosphoglycerate kinase 1 (Pgk1) respectively, and disrupted the biofilm of *C auris*. They all enhanced phagocytosis and host immune response, ultimately significantly reducing the fungal burden in the brain, kidney, and heart of the infected mice. In addition, combinations between mAbs (6h1 and 9F2) or with antifungal agents improved antifungal activity.^[49]^

Similarly, a monoclonal antibody (Anti-HILp MAb) that targeted the Hyr1p/Iff-like proteins of *C auris (Cau*-HILp) from the I to IV branches, and prevented the formation of biofilms. In mice infected with *C auris*, anti-HILp MAb achieved 55% protection against lethal infection, significantly reduced the fungal burden in kidney tissue, and enhanced the phagocytosis and killing ability of macrophages against *C auris*.^[50]^

#### 3.3.2. Proteins inhibitor

Turbinmicin, a secondary metabolite isolated from the marine bacterium *Micromonospora* sp, disrupted *C auris* biofilms by specifically targeting the *Sec14* of *C auris*, impeding vesicle transport and extracellular matrix formation in biofilms. In vitro, the MIC of turbinmicin against planktonic *C auris* was 0.125 µg/mL. In a rat central venous catheter infection model, turbinmicin reduced fungal counts in a dose-dependent manner.^[51,52]^

Tu J et al^[53]^ discovered a novel class of benzoanilide antifungal agents that all blocked the biosynthesis of virulence factors and fungal cell wall by inhibiting GPI and GPI-anchored proteins. One of the most promising compounds, A1, had strong antifungal activity, with MIC ranged from 0.06 to 2.0 µg/mL. In a mouse model of *C auris* infection, A1 significantly reduced renal fungal burden of mice. And it had no obvious toxicity in vitro or in vivo.

#### 3.3.3. Antifungal peptides

The antifungal peptide Cm-p5, and its dimer derivatives (parallel dimer 1 and reverse dimer 2) both showed antifungal activity by inhibiting the biofilm formation of *C auris*. The IC_50_ of dimer 1 against fluconazole-resistant *C auris* was 26.6 µg/mL, and that of dimer 2 was 9.3 µg/mL. Furthermore, both dimers are weakly toxic to mammalian cells.^[54,55]^

LL-37, an antifungal peptide, can cause oxidative stress, disrupt cell membranes and block the cell cycle of *C auris* in the S phase. It displayed strong antifungal effects, with MIC values ranging from 25 to 100 µg/mL and minimum fungicidal concentration (MFC) values ranging from 50 to 200 µg/mL. In addition, LL-37 exhibited synergistic interactions with conventional antifungals, including fluconazole (fractional inhibitory concentration index [FICI]: 0.25–1.13), amphotericin B (FICI: 0.13–0.31), and caspofungin (FICI: 0.13–0.26).^[56]^

#### 3.3.4. Nanoparticles

Both silver nanoparticles (AgNPs) and bismuth nanoparticles (BiNPs) demonstrated potent antifungal and anti-biofilm activity against *C auris* from clades I to IV including multidrug-resistant strains. AgNPs may disrupt biofilms, cellular ultrastructure and metabolism of *C auris*, with MIC values of <0.5 µg/mL. BiNPs may disrupt the structure of fungal biofilms and cellular morphology by exerting antianhydride activity and releasing antimicrobial compounds (bismuth ions and bismuth thiosulfate), with planktonic MIC values ranging from 1 to 4 µg/mL. Additionally, AgNPs are currently used in wound dressings and controlling skin colonization, while BiNPs show promise as disinfectants or novel antifungal agents.^[57–59]^

Ag–Cu–Co trimetallic nanoparticles (Ag–Cu–Co NPs) and Ag–Fe bimetallic nanoparticles (Ag–Fe NPs) had potent antifungal activity against *C auris* and both arrested the cell cycle in the G2/M phase. The MICs of Ag–Cu–Co NPs ranged from 0.39 to 0.78 µg/mL and MFC varied from 0.78 to 1.56 µg/mL, whereas the MICs of Ag–Fe NPs ranged from 0.19 to 0.39 µg/mL and the MFC ranged from 0.39 to 0.78 µg/mL. The hemolysis test performed on equine erythrocytes confirmed that they were safe for in vivo studies, with a hemolysis rate of 0.63% for Ag–Cu–Co NPs and 12.93% for Ag–Fe NPs at MFC concentrations compared to positive control (100%) and negative control (0%).^[60,61]^Therefore, with their intense anti-*Candida* activity and nearly null toxicity nature, Ag–Cu–Co and Ag–Fe NPs were considered as potential alternatives for antifungal chemotherapy against multidrug-resistant (MDR) *C auris* strains.

#### 3.3.5. Repositioning drugs

Gowri M et al^[62]^ demonstrated that sertraline, an antidepressant agent, had antifungal activity, with the MIC of 20 to 40 µg/mL against *C auris* tested. It could disrupt cell membranes of *C auris* by binding to the sterol 14 alpha demethylase to inhibit ergosterol biosynthesis, and could also inhibit fungal biofilm and mycelium formation.

The antimalarial drug mefloquine (MEF) exhibits antimicrobial activity and its derivatives (2450, 4377, 13,480, and 3,05,758) also has similar antifungal activity against *C auris*, with MIC values ranging from 2 to 8 µg/mL against all tested strains. Among these, derivative 4377 displayed the highest efficacy with MIC values of <4 µg/mL. They had multi-target mechanism of action to kill *C auris* including interfering with mitochondrial and vacuolar function, and regulating the expression of fungal virulence factors.^[63]^

Disulfiram, an aldehyde dehydrogenase enzyme inhibitor used for alcohol dependence treatment, was identified as an antifungal agent against *C auris*. It showed superior activity to fluconazole, with MIC values ranging from 1 to 8 µg/mL, and inhibited biofilm of *C auris* by increasing cell aggregation, with BMIC_50_ (the lowest concentration of drug that inhibited at least 50% biofilm formation) values of 32 µg/mL.^[64]^

#### 3.3.6. Derivatives

Srivastava V et al^[65]^ synthesized 6 Piperidine based 1,2,3-triazolylacetamide derivatives (pta1–pta6) and tested their antifungal activity as well as their mechanism of action against clinical *C auris.* They found that pta1, pta2 and pta3 derivatives demonstrated particularly potent activity, with MIC values ranging from 0.24 to 0.97 µg/mL and MFC ranging from 0.97 to 3.9 µg/mL. As with capability of damaging DNA, arresting cell cycle in S phase and inducing cellular apoptosis, these compounds were considered as potential *Candida*tes against *C auris* infections in spite of their lower toxicity profile.

Cinnamaldehyde-based azole derivatives 6f can block the cell cycle of *C auris* in S and G2/M phases, inhibit the activity and expression of antioxidant enzymes, as well as lead to apoptosis of cells. Moreover, the drug sensitivity tests showed that 6f effectively suppressed the growth of *C auris*, with an MIC value of 0.98 µg/mL.^[66]^

A series of novel eugenol tosylate congeners (C1-C6) arrested cell cycle in G0/G1 phase and exhibited potent antifungal activity against *C auris*. C5 had the highest potency (MIC = 0.98 g/mL), coupled with minimal hemolytic activity (0.6–7.3% hemolysis rate), exhibiting extremely low toxicity.^[67]^

### 3.4. Combination of drugs

Multiple resistance of *C auris* to commonly used antifungal agents has hampered treatment of *C auris* infections in recent years, and combination of drugs are promising therapeutic approaches (Table 4).

**Table 4:** Various combinations of drugs against *C auris*.

Class	Combination	*C auris* origin/clades (samples)	Interaction	Source	FICI	References
Combination between antifungal drugs	Caspofungin/isavuconazole	Clades I–IV (n = 23)	SYN (14/23)	In vitro:planktonic isolates	0.03–0.5	^[68]^
	Caspofungin/posaconazole	Clades I–IV (n = 8)	SYN (4/8)	In vitro:planktonic isolates	0.031–2.001	^[69]^
	Micafungin/voriconazole	Clade IV (n = 12)	SYN (4/12)	In vitro:planktonic isolates	0.37–0.49	^[70]^
	Sertraline/voriconazole	Clade IV (n = 12)	SYN (4/12)	In vitro:planktonic isolates	0.37–0.49	^[70]^
	Anidulafungin/amphotericin B	*C auris* CJ94, CJ97, CJ98, CJ99, CJ100, and CJ102 (n = 6, phylogenetically close to Clade III)	SYN (6/6)	In vitro:planktonic isolates	–	^[71]^
	Caspofungin/amphotericin B	*C auris* CJ94, CJ97, CJ98, CJ99, CJ100, and CJ102 (n = 6, phylogenetically close to Clade III)	SYN (6/6)	In vitro:planktonic isolates	–	^[71]^
Combination of antifungal drugs with non-antifungal compounds	Miltefosine/amphotericin B	Clades I–IV (n = 12)	SYN (3/12)	In vitro:planktonic isolates	0.5	^[74]^
	Colistin/caspofungin	*C auris* (India [n = 12], Korea [n = 2], Japan [n = 1], n = 15)	SYN (15/15)	In vitro:planktonic isolates	0.08–0.14107	^[75]^
	Colistin/amphotericin B	*C auris* (CBS 10,913, 12,372, 12,373, 12,766–12,777, n = 15)	SYN (5/15)	In vitro:planktonic isolates	0.1563–0.375	^[76]^
	Colistin/isavuconazole	*C auris* isolates from the collection of Westerdijk Fungal Biodiversity Institute (n = 15)	SYN (14/15)	In vitro:planktonic isolates	0.3125–0.5	^[77]^
	Brilacidin/caspofungin	*C auris* 467/2015–471/2015 (n = 5)	SYN (5/5)	In vitro:planktonic isolates	0.39	^[78]^
	Bud oil formulation/fluconazole	Clades I–IV (n = 10)	SYN	In vitro:planktonic isolates	0.28125	^[79]^
	Bud oil formulation/flucytosine	Clades I–IV (n = 10)	SYN	In vitro:planktonic isolates	0.1875	^[79]^
	Citral/anidulafungin	*C auris* UPV (n = 19)	SYN (7/19)	In vitro:planktonic isolates	0.13–0.77	^[80]^
	Citral/amphotericin B	*C auris* UPV (n = 19)	SYN (8/19)	In vitro:planktonic isolates	0.27–1.48	^[80]^
	Citral/fluconazole	*C auris* UPV (n = 19)	SYN (8/19)	In vitro:planktonic isolates	0.13–2	^[80]^
	Posaconazole/clorgyline analogs M19	Clade I–II (n = 3)	SYN (3/3)	In vitro:planktonic isolates	0.10–0.21	^[81]^
	Voriconazole/clorgyline analogs M19	Clade I–II (n = 3)	SYN (2/3)	In vitro:planktonic isolates	0.25–0.28	^[81]^
	Itraconazole/clorgyline analogs M19	Clade I–II (n = 3)	SYN (3/3)	In vitro:planktonic isolates	0.20–0.30	^[81]^
	Posaconazole/clorgyline analogs M25	Clade I–II (n = 3)	SYN (3/3)	In vitro:planktonic isolates	0.12–0.47	^[81]^
	Voriconazole/clorgyline analogs M25	Clade I–II (n = 3)	SYN (3/3)	In vitro:planktonic isolates	0.24–0.45	^[81]^

n = the number of *Candida auris* samples used in the experiment. Drug interactions are classified as synergistic (FICI < 0.5), additive (FICI ≥ 0.5 but <1), indifferent (FICI ≥ 1 but <4) or antagonistic (FICI ≥ 4).

FICI = fractional inhibitory concentration index, SYN = synergic interaction, UPV = Universitario y Politécnico La Fe (Valencia, Spain).

#### 3.4.1. Combination of antifungal agents

Caspofungin/isavuconazole combination exhibited synergistic effects against 23 isolates of *C auris* from clades I to IV. In planktonic cells, synergy was observed in 60.9% (14/23) of isolates (FICI range: 0.03–0.5). Synergism were also observed with caspofungin and isavuconazole against biofilms (12/14 sessile isolates), the FICI ranging from 0.023 to 0.5. In a mouse model treated with a combination of caspofungin (1 mg/kg) and isavuconazole (20 mg/kg) daily, kidney fungal burden was reduced by more than 3 log volumes compared to control mice (*P* < .001), while monotherapy with caspofungin (1 mg/kg, daily) or isavuconazole (20 mg/kg, daily) were statistically ineffective.^[68]^

Similar synergistic interactions between posaconazole and caspofungin were also observed in an assay against 8 isolates of *C auris* from clades I to IV. In addition to the synergism against planktonic isolates (4/8, FICI range: 0.247–0.49), posaconazole was also able to enhance the activity of caspofungin to inhibit the biofilm of *C auris* (8/8), with FICI ranging from 0.091 to 0.5. And the combination of caspofungin (4 mg/L) with posaconazole (0.03 mg/L) greatly increased the proportion of cell death when compared to untreated, caspofungin-exposed or posaconazole-treated biofilms.^[69]^

More recently, synergy of voriconazole with micafungin or sertraline was evaluated against 12 isolates of *C auris* from clades IV. The micafungin/voriconazole combination has similar antifungal activity with the sertraline/voriconazole combination (both were active in 4/12, FICI range 0.37–0.49) in vitro, and no antagonism was found in any of the combinations.^[70]^

The combination of amphotericin B (0.5 mg/L) with either anidulafungin or caspofungin (2 mg/L) produced a rapid synergistic effect with enhanced fungistatic/fungicidal activity. In addition, ≥0.5 mg/L of anidulafungin or caspofungin in combination with 1 mg/L of amphotericin B achieved a fungicidal effect against all tested isolates of *C auris*.^[71]^

#### 3.4.2. Combination of antifungal agents with non-antifungal compounds

Miltefosine, an antileishmanial agent, demonstrates notable antifungal activity both in vivo and in vitro.^[72,73]^ When miltefosine was used alone, the MIC against a dozen *C auris* from clades I to IV was 2 µg/mL, and miltefosine combined with amphotericin B had synergistic effect on 3 of 12 isolates of *C auris* (FICI = 0.5).^[74]^

Colistin monotherapy was ineffective against *C auris*, with MIC > 64 mg/mL, but synergistic effects were observed against 15 isolates of *C auris* in vitro when paired with caspofungin (FICI range: 0.08–0.14107) or amphotericin B (FICI range: 0.1563–0.375) or isavuconazole (FICI range: 0.3125–0.5).^[75–77]^

Combinations of the small molecule host defense peptide mimetic brilacidin (BRI) with caspofungin combination also exhibited synergism against *C auris*, with FICI of 0.39. And the combination of 10 µM BRI and 0.125 µg/mL CAS inhibited about 95% metabolic activity of *C auris* 467–470/2015 and 474/2015.^[78]^

Some essential oils and their constituents, have shown anti-*Candida* activity.^[79]^ One of the most effective essential oils was extracted from clove bud. Formulations of clove bud oil with fluconazole (FICI = 0.28125) and flucytosine (FICI = 0.1875) displayed synergistic interactions.^[79]^ Similarly, essential oils citral (3,7-dimethyl-2-6-octadienal) is a terpene mixture of 2 geometric isomers (the geranial or trans-citral and the neral or cis-citral). The researchers assessed the combined activity of citral paired with anidulafungin, amphotericin B, or fluconazole against 19 *C auris* isolates. The combination of 0.06 µg/mL anidulafungin and 64 µg/mL citral demonstrated optimal efficacy, with a survival rate of 63.2% in *Caenorhabditis elegans* infected with *C auris*. Fluconazole/citral combination reduced the MIC of fluconazole from >64 to 1 to 4 µg/mL, and effectively reduced mortality in *C elegans*. However, amphotericin B/citral combination was only effective in vitro.^[80]^ These essential oils and their combinations with antifungal agents may provide useful options for the disinfection and treatment of *C auris* infections.

Clorgyline had previously been shown to be a multi-target inhibitor of Cdr1 and Mdr1 efflux pumps of *Candida albicans* and *Candida glabrata*. In a recent study, of 6 Clorgyline analogs, M19 and M25 were identified as potential sensitizers of azole resistant strains. The combinations of M19 and M25 with azoles (posaconazole, voriconazole or itraconazole) had synergistic effect on the drug-resistant *C auris* clade I isolates, with FICI ranging from 0.10 to 0.47.^[81]^

### 3.5. IPC measures

Against *C auris* infection, prevention is also an important and effective health strategy. Surveillance studies have shown that *C auris* can survive for at least 2 weeks on wet or dry surfaces, for at least 7 days on contaminated fabrics, and for several months on the surface of patient skin.^[82–85]^ The remarkable environmental stability of *C auris* necessitates rigorous infection control measures in patient management, healthcare worker protection, and institutional hygiene practices.

#### 3.5.1. Patients

Once *C auris* is identified from a non-quarantining patient in a health care setting, the patient and close contacts should be screened until microbiological diagnostic results are available, and point-prevalence surveys of entire wards or facilities should be conducted to assess the extent of transmission and identify all colonized patients.^[86,87]^ Patients with confirmed *C auris* colonization or infection should be quarantined as individuals, screened weekly until discharge, and labeled for at least 1 year after the first negative screening culture.^[88–90]^

#### 3.5.2. Healthcare workers

Healthcare professionals should pay more attention to the prevention and control of *C auris* infection and strictly implement IPC measure.^[91,92]^ Staff and visitors should wear gowns, gloves, and medical masks when entering the patient’s room. Upon exiting, staff and visitors must be properly removed, followed by immediate hand hygiene using alcohol-based hand rub.^[93]^

#### 3.5.3. Medical institutions

Targeted environmental cleaning should prioritize high-touch surfaces in close proximity to colonized or infected patients, including bed rails, bedside tables, nurse call buttons, and patient monitoring equipment. Particular attention must also be paid to medical devices such as IV poles, ventilators, and mobile consoles. Additionally, “wet” or shared areas like sinks, faucets, and bathroom surfaces (e.g., toilets, floors) are common sites for contamination and require rigorous disinfection. Reusable patient-care equipment, including shared thermometers, blood pressure cuffs, and linens, poses a significant cross-transmission risk and must be meticulously cleaned and disinfected between uses. Critically, these efforts must employ hospital-grade disinfectants proven effective against *C auris*. For surface disinfection of medical devices, the CDC recommends the use of List P or List K disinfectants of US Environmental Protection Agency (EPA), including disinfection products composed of chemicals such as hydrogen peroxide (H_2_O_2_), sodium hypochlorite and peracetic acid.^[94,95]^ In addition, chlorhexidine, ultraviolet light (UV), and H_2_O_2_ can be used to decontaminate and disinfect the environment and equipment.^[96–99]^

Chlorhexidine bathing is routinely routinely employed in hospital settings to reduce colonization and infection by multidrug-resistant fungus. Daily bathing with 2% chlorhexidine significantly reduces the skin fungal burden of *C auris*, particularly in high-risk folds such as the peri-auricular area, axillae and groin, thereby reducing the risk of transmission to the environment and other patients. However, routine chlorhexidine bathing still doesn’t stop the spread of *C auris*.^[100,101]^ In some studies, the addition of commonly used topical compounds has been shown to improve the effect of chlorhexidine. Fluconazole and chlorhexidine had a strong synergistic effect in inhibiting biofilm growth of *C auris* with FICI < 0.1875.^[102]^ Isopropanol, tea tree oil and lemongrass oil were also found to enhance the activity of chlorhexidine against *C auris*. Moreover, chlorhexidine formulated with a new advanced performance technology formulation (APT-CH) containing terbinafine or clotrimazole was more effective in inhibiting *C auris* than traditional chlorhexidine.^[103]^ For mucosal and gastrointestinal decolonization of *C auris*, the novel antifungal agents ibrexafungerp and fosmanogepix are considered the most promising candidates due to their unique mechanisms of action and oral bioavailability. They represent a potential strategy for eradicating the fungus from sanctuary sites like the gastrointestinal tract. However, this application remains under clinical investigation and is not yet established as standard therapy.

Ultraviolet-C light (UV-C) can disinfect the surfaces missed or under-covered by manual disinfection to reduce the contamination of *C auris* to the environment and can be used as an adjunct to standard cleaning and disinfection of *C auris*. However, the disinfection effect of UV-C is affected by many factors such as distance, exposure time, susceptibility of *C auris* clades and line of sight.^[104–107]^ A mobile 254 nm UV-C tower equipped with high-performance bulbs could achieves >99.97% inactivation of *C auris* within 7 minutes, improving disinfection efficiency.^[108]^

Studies have shown that the antifungal effect of H_2_O_2_ against *C auris* enhanced with the increase of H_2_O_2_ concentration. A micellar H_2_O_2_-based aqueous disinfectant (mH_2_O_2_) killed *C auris* biofilms within 15 minutes at 5% concentration and within 3 minutes at 10% concentration. And EPA-registered H_2_O_2_-based disinfectant towelettes were also effective against *C auris*.^[109]^

Sporicidal disinfectants, including electrolyzed water, sodium dichloroisocyanurate, as well as peracetic acid/H_2_O_2_, can also effectively clean the surfaces of medical facilities, and novel application methods such as electrostatic sprayers can improve disinfection effect.^[110]^

Successful containment of *C auris* outbreaks has been documented in healthcare facilities across Saudi Arabia, South India, Germany, and the United States through rigorous implementation of IPC measures.^[111–114]^ These successful cases showed that IPC measures were feasible for the prevention of *C auris* outbreaks.

## 4. Conclusions and future perspectives

MDR *C auris* can survive for long periods in the environment and can be transmitted rapidly in the population, leading to high mortality. And the increasing multi-resistance of *C auris* to first-line antifungal agents has reduced the treatment options around the world, making clinical antifungal treatment and prevention a great challenge. Given its potential for independent and simultaneous emergence across different regions, the development of novel and effective therapeutic strategies is urgently needed.

First, several antifungal agents (e.g., opelconazole, VT-1598, rezafungin, ibrexafungerp, fosmanogepix, manogepix, and T-2307) in clinical development have displayed a favorable safety and tolerability profile in clinical trials, and they may stop the evolution of multidrug-resistant *C auris*, reduce adverse effects of drug as well as improve the cure rates of patients. However, clinical trial data specifically targeting *C auris* infections remain limited, with some results yet to be published. Consequently, more clinical trials are still needed to validate the effectiveness of these drugs in clinical practice.

Second, novel antifungal agents have been shown to be potent in vitro activity against *C auris*. In recent years, with a broader understanding of the virulence and resistance mechanisms of *C auris*, some new targets against *C auris*, such as *Sec14p*, can be developed corresponding targeted drugs. And researchers synthesized or screened compounds with antifungal activity, including nanoparticles and repurposed drugs like sertraline. Additionally, immunomodulatory approaches, such as monoclonal antibodies (mAbs), represent an emerging strategy to combat *C auris* infections. Combination of drugs (e.g., caspofungin/ amphotericin B, micafungin/voriconazole, and fluconazole/chlorhexidine acetate) may act synergistically to enhance their efficacy, have been suggested as an alternative option for the treatment of *C auris* infections. Although some reliable experimental data and results for drugs against *C auris* have been obtained, most of the drugs have only been evaluated in vitro, and their effectiveness needs to be further assessed in animal and clinical trials. In addition, majority of experimental studies were based on a limited number of isolates samples and did not include all clades of *C auris*. Data and results obtained from different clades or strains were controversial in some cases. Thus, further research is needed to optimize these therapeutic strategies.

Third, in addition to developing treatments, improving the recognition of the risk factors and clinical features of *C auris* infection is critical for timely diagnosis and effective clinical management. Controlling the source of infection, managing the transmission route, protecting the susceptible population, as well as improving the awareness and vigilance of medical staff about *C auris* will help to reduce the incidence of *C auris* infection. The World Health Organization, the Center for Disease Control and Prevention, and the EPA have developed guidelines for isolating patients properly, educating health care workers about hospital operations, disinfecting medical equipment, and using appropriate disinfectants.

Finally, the emergence of *C auris* underscores the persistent threat of multidrug-resistant pathogens in healthcare settings. To mitigate outbreaks, countries must implement real-time surveillance systems and develop comprehensive response strategies, including rapid diagnosis, reporting, isolation, and evidence-based treatment protocols.

Overall, addressing the challenge of *C auris* requires a coordinated global effort involving clinicians, researchers, healthcare institutions, and policymakers.

## Author contributions

**Conceptualization:** Jieyu Zhang, Lichao Zhong, Fei Cao.

**Formal analysis:** Chunxiang Chen, Licong Ye, Hua Xia, Yi Hong.

**Writing – original draft:** Jieyu Zhang, Lichao Zhong, Fei Cao.

**Writing – review & editing:** Fei Cao.
